# Ultrastructural Analysis of Johnston’s Organ and Brain Organization in *Philaenus spumarius* (Hemiptera: Aphrophoridae)

**DOI:** 10.3390/insects17010015

**Published:** 2025-12-22

**Authors:** Milos Sevarika, Christoph Kleineidam, Roberto Romani

**Affiliations:** 1 Department of Agricultural, Food and Environmental Sciences, University of Perugia, 06123 Perugia, Italy; roberto.romani@unipg.it; 2 Department of Biology, University of Konstanz, 78464 Konstanz, Germany; christoph.kleineidam@uni-konstanz.de

**Keywords:** neuroanatomy, Micro-CT, morphology, confocal, antennae, *Xylella fastidiosa*

## Abstract

Insects rely on a variety of sensory cues for orientation, with antennae playing a central role in receiving and transmitting information about the environment. *Philaenus spumarius* (Hemiptera: Aphrophoridae), a spittlebug and vector of the bacterium *Xylella fastidiosa*, has a reduced number of antennal sensilla, yet demonstrates effective multimodal communication through olfactory and vibrational signals. This study aimed to investigate how the simplified sensory system of *P. spumarius* relates to the primary neuropils of the brain. We examined the ultrastructural organization of Johnston’s organ using scanning and transmission electron microscopy, complemented by previous data on antennal sensilla. Brain organization was investigated by Micro-CT and confocal laser scanning microscopy, which enabled us to identify the primary neuropiles. In addition, we conducted antennal and single sensillum backfills to trace sensory neurons to the brain. Our findings provide insight into the adaptation of a simplified sensory system for effective communication and orientation in *P. spumarius*.

## Simple Summary

The spittlebug *Philaenus spumarius* is a key vector of a plant-pathogenic bacterium and mostly depends on its antennae to detect environmental and inter/intraspecific stimuli. Compared with many other insects, *P. spumarius* shows a reduced number of antennal sensory structures, but despite of this, it can efficiently orient and communicate. This study investigates how such a simplified sensory system is reflected in the organization of its brain by examining the fine structure of Johnston’s organ and analyzing the main regions of the brain. We also traced the pathways of antennal nerves up to the central nervous system. The results show how *P. spumarius* integrates limited sensory input through a neural organization that supports effective communication and movement. These findings provide insight into how insects can adapt their nervous systems to different sensory abilities, giving a basic knowledge for further behavioral and chemical ecology studies of this harmful pest.

## 1. Introduction

Insects orient themselves in the environment using different sensory modalities (i.e., chemical, mechanical, physical) [1,2,3,4], mediated by different stimuli that are primarily detected by the antennae, considered the insect’s primary sensory organs [5]. The functional units responsible for perceiving these stimuli are the sensilla, i.e., minute functional organs capable of detecting specific stimuli. Given the extraordinarily high diversity of insects and the variety of stimuli on which they rely throughout their lives, sensilla are adapted to specific functions. In chemoreceptors, pores are usually located at the sensillum tip or scattered on the sensillum cuticular wall [6,7]. The sensilla sensory neuron axons project to the central nervous system and terminate at specific neuropiles [8,9,10,11].

In addition to externally visible sensilla, insects possess sensilla present in the internal lumen of various organs/body parts, including legs, wings, antennae, abdomen, and tympanal organ. These internal sensilla are known as scolopidia and are particularly prominent in the insects’ antennae [12]. In the second antennal segment, the pedicel, scolopidia are arranged in a circular pattern, forming a specialized structure called Johnston’s organ (JO) [12,13,14]. Scolopidia are extremely sensitive organs, capable of responding to the slightest antennal movement. The degree of their sensitivity was recorded in *Toxyrhynchites brevipalpis* females, which responded to antennal deflections of ±0.0005° induced by ±11 nm air particles [15]. To date, Johnston’s organ has been associated with various functions, from gravireception to sound perception, flight control, airflow detection, and vibrational sensing [16,17,18].

The axons of all antennal sensory organs are merged into the antennal nerve and project to the primary neuropiles in the brain. Different neuropiles are specialized for processing specific sensory signals. For instance, visual signals from eyes are processed in the optic lobes (Ols), mechanical signals are analyzed in the antennal mechanosensory and motor center (Ammc), whereas the olfactory signals are primarily analyzed in the antennal lobes (Als). The Als, considered as the primary olfactory centers of insect’s brain, are organized into small spherical functional units, called olfactory glomeruli. The number of glomeruli ranges from fewer than 10 up to several hundred, and each glomerulus receives inputs from the olfactory sensory neurons that express the same type of olfactory receptors [9,11,19]. Olfactory information is subsequently modulated by local interneurons, and relayed to higher brain regions via projection neurons. Projection neurons convey information to the higher olfactory centers, including lateral horn and the mushroom bodies [10,11,20,21].

Sensory organs are adapted to specific ecological needs, resulting in their better performance (i.e., finding a food source, mate, etc.). This is especially evident in insects that have developed macroglomeruili in the antennal lobe, which are common in insects that use pheromones for long-range communication. However, it has been shown that macroglomeruli are also associated with food odors [22,23]. The changes in glomeruli size are primarily associated with the number of olfactory sensory neurons (OSNs) they receive [20,24] and the number of synapses [25]. In addition to differences in the size of individual glomeruli, significant variations have been observed in the number of glomeruli between various species. In most species, the Al is composed of approximately 50–200 glomeruli [26,27,28,29,30]. However, extreme values were found in some species, as in the case of locust, which shows between 1000 and 3000 glomeruli [31,32]. In contrast, species like aphids [33,34], dragonflies [35], planthoppers and leafhoppers [36] either lack glomeruli or only have a very low number. Moreover, variations were observed in the mushroom body development, being larger in social insects that display non-stereotyped behavioral responses [37].

*Philaenus spumarius* L. (Hemiptera: Aphrophoridae), commonly known as the meadow spittlebug, is a species that has received increasing attention in recent years due to its role as the primary vector of *Xylella fastidiosa*, a destructive xylem-limited bacterium currently spreading across Italy and Europe [38]. Beyond its characteristic xylem-feeding behavior and spittle mass production, *P. spumarius* is characterized by having a significantly reduced number of antennal sensilla [39]. A recent investigation of *P. spumarius* antennae revealed the presence of only three olfactory sensilla (sensilla basiconica), each one housing 21 OSN [39]. Despite this limited number of olfactory sensilla, *P. spumarius* has shown positive responses to a variety of chemical compounds from plants and conspecifics in laboratory behavioral bioassays [40,41,42,43,44]. In addition to olfactory cues, *P. spumarius* utilizes vibrational signals for communication. The vibrational repertoire of *P. spumarius* has been characterized, revealing the use of vibrational signals in intraspecific sexual communication [45]. Thus, *P. spumarius* relies on a multimodal communication system.

This study aimed to investigate how the simplified sensory system of *P. spumarius* antennae correlates with brain development. To do this, we first investigated the ultrastructural organization of Johnston’s organ by scanning and transmission electron microscopy. By Micro-CT and confocal laser scanning microscopy, we studied the brain organization and identified the primary neuropiles. Moreover, we conducted antennal and single sensilla backfills, enabling us to investigate the target regions within the *P. spumarius* brain.

## 2. Materials and Methods

Adults of *P. spumarius* were collected during 2020 and 2021 on meadows near the University of Konstanz (Konstanz, Germany) and in the Umbria region (Italy). Once captured, insects were transferred to the rearing facility. Insects were reared in mesh cages (Kweekkooi 40 × 40 × 60 cm, Vermandel, Hulst, The Netherlands) under controlled conditions (25 ± 2 °C, L16:D8, RH 60 ± 5%). Fresh *Vicia faba* L. plants were placed inside the cage as a food source and replaced every 10 days.

### 2.1. Scanning Electron Microscopy

Scanning electron observations were conducted on 10 individuals, which were anesthetized by low-temperature exposure (−18 °C for 2 min) and placed in 50% ethanol. To observe antennae in a natural position, the whole head with the antennae was detached from the rest of the body. Moreover, to observe the antennae from all sides, they were removed from the head by a fine scalpel blade under a stereomicroscope. The prepared specimens were dehydrated in a series of graded ethanol (60, 70, 80, 90, 95, and 99%), each step for 15 min. After dehydration, specimens were submerged in pure HMDS (Hexamethyldisilazane, Sigma-Aldrich, Gillingham, Dorset, UK) and allowed to dry under the hood, at room temperature. Samples were mounted on aluminum stubs, and to obtain a clear view of the different sides, the specimens were positioned with different orientations. Mounted specimens were gold-sputtered using a “Balzers Union^®^ SCD 040” unit (Balzers, Vaduz, Liechtenstein). The observations were carried out using a FE-SEM Zeiss^®^ SUPRA 40 (Carl Zeiss NTS GmbH, Oberkochen, Germany) and a Philips^®^ XL 30 (Eindhoven, The Netherlands) operating at 7–10 KV, WD 9–10 mm, and analyzed by a SMART-SEM^®^ (v6.05, Carl Zeiss Microscopy GmbH, Germany) software.

### 2.2. Light and Transmission Electron Microscopy

Ten individuals were anesthetized by exposure to low temperature (−18 °C for 1 min). Immediately after, they were immersed in a solution of 2% glutaraldehyde and 2.5% paraformaldehyde in 0.1 M cacodylate buffer + 5% sucrose, pH 7.2–7.3. Complete antennae were detached from the head and left at 4 °C for 2 h. The specimens were kept at 4 °C overnight in the same buffer, then the specimens were post-fixed in 1% OsO4 (osmium tetroxide) for 1 h at 4 °C and rinsed in the same buffer. Later, the specimens were dehydrated in a series of graded ethanol from 60 to 99% and embedded in Epon-Araldite with propylene oxide as the bridging solvent. Thin sections were taken with a diamond knife on a LEICA ULTRACUT R ultramicrotome (Leica^®^, Wetzlar, Germany) and mounted on formvar-coated 50 mesh grids. Then, sections on grids were stained with uranyl acetate (20 min, room temperature) and lead citrate (5 min, room temperature). Finally, the sections were investigated with a Philips^®^ EM 208 (Amsterdam, The Netherlands). Digital pictures (1376 × 1032 pixels, 8b, uncompressed greyscale TIFF files) were obtained using a high-resolution digital camera MegaViewIII (SIS^®^, Münster, Germany) connected to the TEM. Semithin and thin cross sections taken at the joint level between the arista and the pedicel were used to count scolopidia.

### 2.3. Histology and Immunocytochemistry

Live *P. spumarius* individuals were anesthetized by placing them in a freezer for 2 min. Using fine tweezers, brains were carefully dissected and immediately placed in freshly prepared 4% paraformaldehyde (PFA, Electron Microscopy Science, Hatfield, PA, USA) dissolved in 0.01 M phosphate-buffered saline solution (PBS) for fixation. The samples were kept at room temperature on a shaker for 1 h. After fixation, the brains were washed 7 times for 15 min with PBS containing 1% Triton X-100 (PBS-Tx, Sigma Aldrich, St. Louis, MO, USA). The brains were preincubated overnight in an antibody blocking solution. Subsequently, the brains were incubated with primary antibody, monoclonal anti-mouse Synapsin 1 (Hybridoma Bank, Iowa City, IA, USA, SYNORF1) at a 1:30 dilution for 3 days at room temperature on a shaker. After primary antibody incubation, the brains were rinsed 8 times for 30 min with PBS-Tx and incubated for 3 days with the secondary goat anti-mouse conjugated to Alexa Fluor 546 antibody (Thermo Fisher Scientific, Waltham, MA, USA) diluted 1:500. To visualize cell nuclei, DAPI (Sigma-Aldrich, Darmstadt, Germany) at a dilution of 1:500 was added simultaneously with the secondary antibody. After antibody incubations, the brains were washed 11 times for 20 min with PBS-Tx and dehydrated in an ethanol series of increasing concentrations (50, 75, 95, and twice in 100%) for 30 min each step. Following dehydration, the brains were treated with xylene for 2 min and embedded in DPX mounting medium (Sigma-Aldrich, USA) between two cover slips separated by a custom-made metal spacer.

### 2.4. Antennal Backfills

Insect were anesthetized for 2 min in a freezer and immobilized on a cover glass. The antennae were positioned vertically and secured using Patafix, which was shaped into a wall around the scape. Within this Patafix wall, one µL of 4% Neurobiotin (Neurobiotin Plus, Vector Laboratories, Newark, CA, USA) was applied, after which the flagellum was removed by a razor. The insects were then placed in a dark chamber for 2 h with a piece of wet paper to maintain high humidity. Brain dissection and fixation were carried out as described above. Synapsin-rich neuropiles were stained with a monoclonal anti-mouse Synapsin 1 was applied at 1:30 dilution followed by 8 washes with PBS-Tx for 30 min each. The brains were subsequently incubated with Cy3 conjugated Streptavidin (Jackson ImmunoResearch, West Grove, PA, USA) at a 1:400 dilution, goat anti-mouse conjugated to Alexa Fluor 488 (Thermo Fisher Scientific, USA, 1:500) and DAPI (Sigma-Aldrich, Germany, 1:500) for 3 days. Subsequently, the brains were washed 11 times in PBS-Tx for 20 min each, dehydrated in graded series of ethanol, cleared in xylene and mounted in DPX, as reported above.

### 2.5. Single Sensillum Backfills

Insects were immobilized on a cover glass with ventral side facing upward using dental wax. A thin layer of the dental wax was applied on the antennal ledge, on which the pedicel was attached. To facilitate the approach of the glass electrode to the sensilla basiconica, the arista was halved using micro scissors. The prepared specimens were positioned under a light microscope (Examiner A1, Zeiss, Oberkochen, Germany) equipped with a 250× magnification objective lens. A glass electrode, prepared using a micropipette puller (Sutter instrument, P-2000, Novato, CA, USA), was connected to a frequency generator via a piezo element and mounted on a micromanipulator. When the electrode was positioned near the sensillum basiconica, the frequency was adjusted so that the glass electrode started to vibrate, causing the sensillum to break. Immediately afterward, 1 µL of 4% Neurobiotin (Neurobiotin Plus, SP-1150; Vector, Newark, CA, USA) was applied over the broken sensillum. The specimens were then placed in a dark box for 2 h. Subsequently, brains were dissected and fixed as previously described. To visualize neurobiotin, Alexa Fluor 488 conjugated streptavidin was applied (Thermo Fisher Scientific, 1:400), whereas for neuropile identification, monoclonal anti-mouse synapsin 1 followed with secondary goat anti-mouse conjugated to Alexa Fluor 546 antibody.

### 2.6. Confocal Laser Scanning Microscopy

Whole-mount preparations were scanned with a Zeiss LSM 510 or a Zeis LSM 880 confocal laser scanning microscope, equipped with a 10× and 20× objective lenses. Images were acquired in sequential mode, utilizing three different excitation wavelengths simultaneously (405 nm for DAPI, 488 nm for Alexa Fluor 488 and Alexa Fluor 488 streptavidin, 540 nm for Cy3, and 560 nm for Alexa Fluor 546). These wavelengths allowed for the visualization of antisynapsin staining, antennal nerve fill and cell body staining. Images were taken at intervals of 0.5 or 1 µm.

The 3D reconstruction of identified neuropiles was performed using Amira software (Amira 5.3, Visage Imaging, Fürth, Germany). Neuropiles were labelled using the segmentation editor with the interpolation option and were subsequently manually verified for accuracy. The final labels were rendered using the SurfaceGen tool.

### 2.7. Micro Computed Tomography (Micro-CT)

Individuals of *P. spumarius* were anesthetized by exposure to low temperatures (−20 °C) for two minutes and immersed in a solution of 2% glutaraldehyde and 2.5% paraformaldehyde in 0.1 M cacodylate buffer +5% sucrose, pH 7.2–7.3 for 24 h at 4 °C. Following fixation, specimens were washed with the same buffer two times for 15 min and stored in 99% ethanol. Specimens were subsequently stained with Lugol’s iodine solution for one week at 4 °C, then washed two times for 15 min with 99% ethanol, mounted in a pipette tip filed with 99% ethanol and sealed with the parafilm. Micro-CT scanning was performed using a Bruker-SkyScan 1172 (Bruker, Billerica, MA, USA) with a voltage of 60 kV and an amperage of 167 uA, a 360° rotation and step size of 0.15°. The resulting image projections were processed with NRecon (Version 1.7.4.6) and exported as an 8-bit BMP image series. The image series were subsequently transformed into a single 8-bit TIFF file. Segmentation was carried out in Dragonfly 2022.2 (Comet Technologies, Montreal, QC, Canada). To ensure uniformity in the segmentation, an OTSU thresholding was applied. The cuticle, brain, and retina were pre-segmented by manually annotating every 25th slice. Regions of interest (ROIs) were exported as a single grayscale TIFF image, where each ROI was assigned a unique label (1–3) and everything else was labelled as background (0). Both the greyscale image and Micro-CT dataset were uploaded to Biomedisa, an online platform for semi-automatic segmentation. The resulting segmentations were imported back to Dragonfly, where ROIs were extracted and compared to assess the segmentation accuracy. Lastly, areas with spilling into the wrong ROI were manually corrected, smoothed, and rendered in 3D using Dragonfly.

## 3. Results

### 3.1. Antenna General Morphology

**Figure 1:**
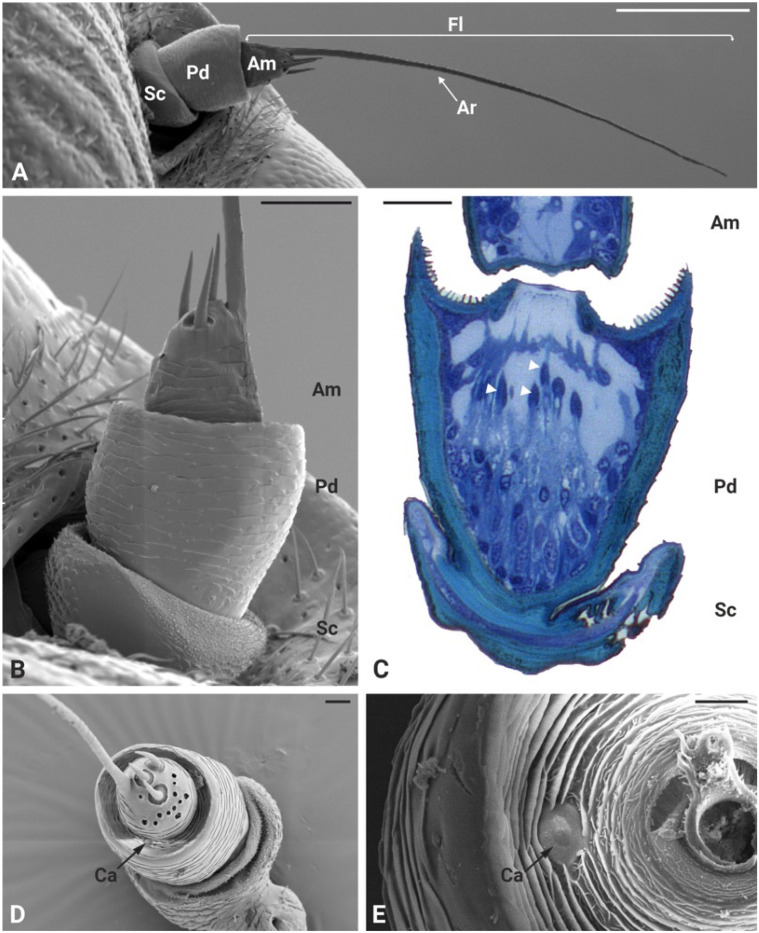
Scanning and light microscopy micrograph of *P. spumarius* antennae. (**A**) Ventral view of the antenna showing a short scape (Sc), a large pedicel (Pd) and the flagellum (Fl), which is composed of a basal ampulla (Am) and a long arista (Ar). (**B**) Close-up view of the basal part of the antenna. (**C**) Longitudinal LM section of the pedicel, showing several scolopidia (white arrowheads) belonging to the Johnston’s organ. (**D**) Frontal view of the antennae showing sensory structures present on the ampulla and the area between the ampulla and the apical part of the pedicel. A campaniform sensillum (Ca) can be observed at the apical part of the pedicel. (**E**) Higher magnification image of the Ca sensillum. Scale bars: (**A**) = 200 μm, (**B**) = 50 μm, (**C**) = 25 μm, (**D**) = 20 μm, (**E**) = 10 μm.

**Figure 2:**
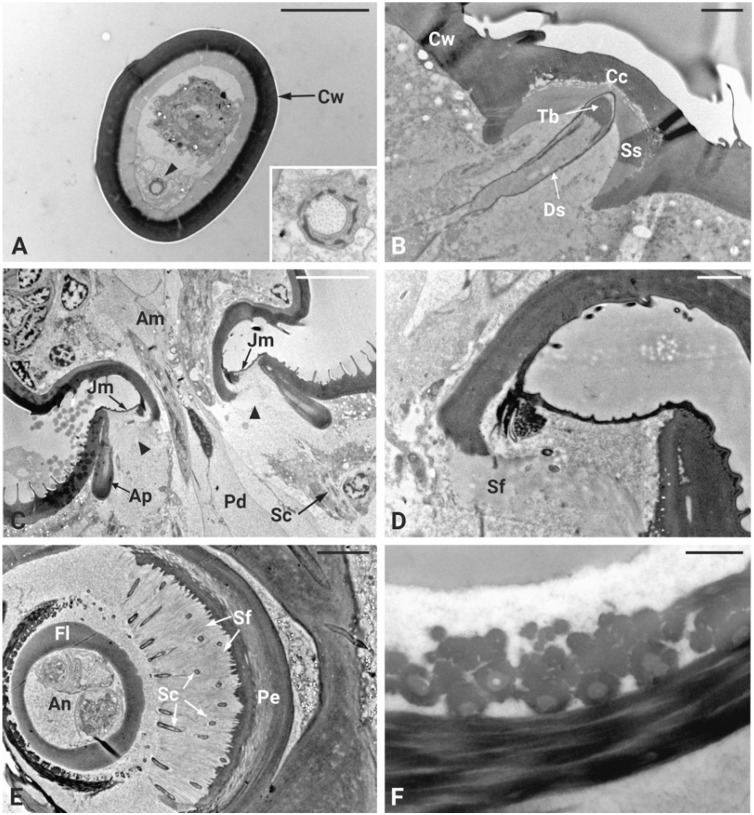
Ultrastructural organization of *P. spumarius* antennae. (**A**) Cross-section of the middle portion of the arista showing an electron-dense cuticular wall (Cw): on one side, a single sensory neuron (arrowhead) surrounded by a dendrite sheath is present. Inset: higher magnification image of the sensory neuron. (**B**) Longitudinal section of sensilla campaniformia showing the cuticular cap (Cc) inserted on the thicker antennal cuticular wall (Cw). A single sensory neuron surrounded by the dendrite sheath (Ds) enters the sensillum lumen and ends just below the Cc. The apical tubular body (Tb) is clearly visible. The sensory neuron is positioned below the cuticular cap and surrounded by a socket septum (Ss). (**C**) Longitudinal section at the joint level between the pedicel (Pd) and the ampulla (Am) of the flagellum. The joint membrane (Jm) extends over the apical part of the pedicel and attaches to the flagellum. The flagellum is inserted on the suspension fibers (arrowheads) that connect to the apodeme (Ap). At the central lumen of the pedicel, a series of scolopidia (Sc) are visible. (**D**). Higher magnification image of the suspension fibers (Sf). (**E**) Cross section of the apical part of the pedicel with the basal flagellum (Fl) at its center. The antennal nerve (An) occupies a large portion of the flagellum’s lumen. The scolopidia (Sc) of Johnston’s organ are distributed in a circular pattern around the flagellum. Individual scolopidia are embedded in the suspension fibers (Sf) between the pedicel (Pe) and ampulla. (**F**) High magnification image of the scolopidia attachment to the flagellum. Scale bars: (**A**) = 5 μm, (**B**) = 2 μm, (**C**) = 10 μm, (**D**) = 2 μm, (**E**) = 5 μm, (**F**) = 0.5 μm.

In *P. spumarius*, the antennae were composed of three segments: the scape, the pedicel and the flagellum. The flagellum was made up of a basal bulbous structure called the ampulla and a long, thread-like segment known as the arista (Figure 1A,B). The antennae were about 800 μm long and 110 μm in diameter. No sexual dimorphism was observed in the number or positioning of the sensory structures. The ampulla contained three sensilla basiconica and twelve sensilla coeloconica, as previously described in detail by [39] (Figure 1D). The arista was long and tapered, often displaying a broken tip. Ultrathin cross-sections of the medial region of the ampulla revealed a single sensory neuron encased in electron-dense material (Figure 2A inset). The ampulla was inserted into the central area of the pedicel. The pedicel was notably enlarged, with a prominent external ridge on its apex, forming a concave area when observed from above (Figure 1C,D). In the space between the pedicel and the ampulla, a campaniform sensillum was observed (Figure 1E). The ultrastructure of the campaniform sensillum followed the characteristic morphology of this type of sensillum, with a thick cuticular wall slightly raised above the antenna cuticle, forming a cap-like structure. Beneath this cuticular cap, a single sensory neuron was observed, ending in a tubular body in central position and close to the internal area of the cuticular cap. The neuron was surrounded by a fine dendritic sheath and was positioned within a socket-like septum (Figure 2B).

### 3.2. Johnston’s Organ Organization

**Figure 3:**
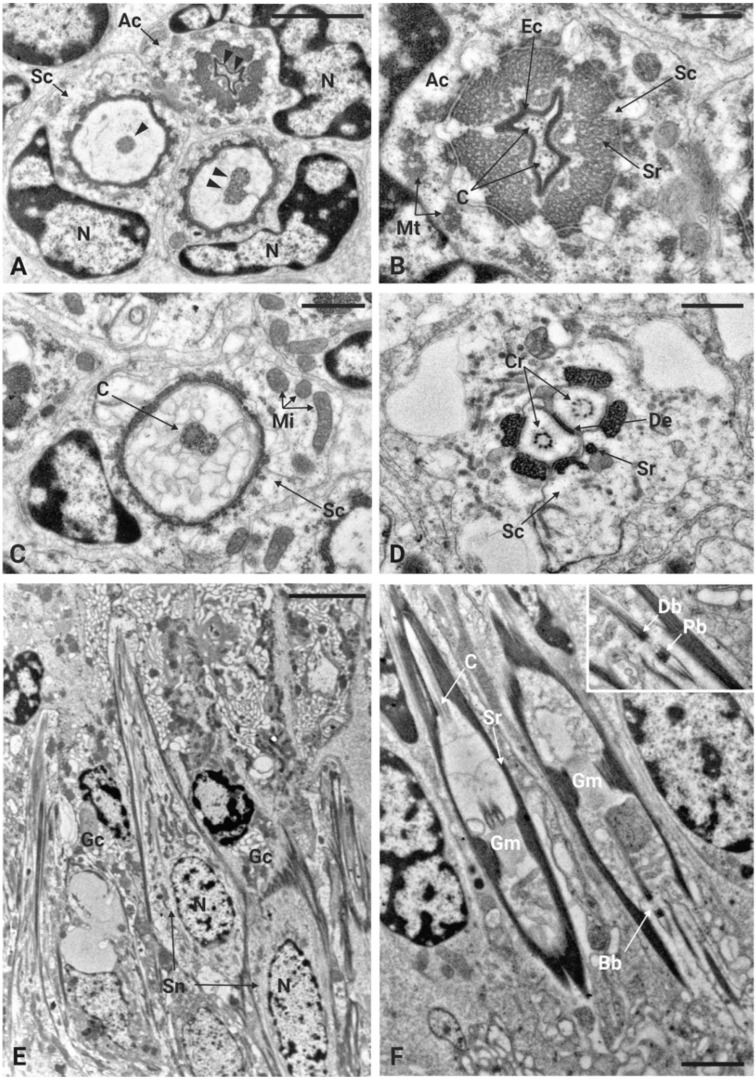
Ultrastructural organization of the scolopidia of Johnston’s organ in *P. spumarius.* (**A**) Cross-section of three scolopidia, each containing a different number of cilia (arrowheads). Two groups of cilia are enveloped by a scolopale cell (Sc), which exhibits a large nucleus (N). The third and more distal group of cilia is enveloped by the attachment cell (Ac). (**B**) Distal part of a scolopidium showing two cilia (C) surrounded by an extracellular cap (Ec). The scolopale rods (Sr) are almost fused at this stage and the scolopale cell (Sc) is significantly reduced. The attachment cell (Ac) surrounds the whole structure and presents densely packed bundles of cytoplasmic microtubules (Mt). (**C**) More proximal section of the scolopidium showing two almost fused cilia. Some mitochondria (Mi) are visible in the cytoplasm of the scolopale cell (Sc). (**D**) Sections taken at the basal part of the scolopidium showing the ciliary roots (Cr) and desmosome (De). Within the scolopale cell (Sc), six segments of scolopale rods (Sr) are visible. (**E**) Longitudinal section taken at the basal part of the pedicel showing the soma of sensory neurons (Sn) associated with the scolopidia with large nuclei (N). Nuclei belonging to glia cells (Gc) are also visible. (**F**) Longitudinal section of scolopidia surrounded by scolopale rods (Sr). Within the rods, a cilium (C) is visible at its proximal part, while at the distal part, the basal bodies (Bb) and granular material (Gm) are present. Inset: high magnification image of the proximal (Pb) and distal (Db) basal bodies. Scale bars: (**A**,**B**) = 1 μm; (**E**) = 5 μm; (**C**,**F**) = 2 μm; (**D**) = 0.5 μm.

TEM investigations of the antenna of *P. spumarius* revealed details of the organization at the level of the flagellum-pedicel joint. The flagellum was inserted into the pedicel through a flexible joint membrane (Figure 2C). At this level, a thin epicuticular layer apparently originating from the epicuticle of the flagellum and pedicel surrounds the base of the flagellum (Figure 2D). The cuticular wall of the proximal part of the flagellum ends within the pedicel lumen, where it is connected to the pedicel itself through a layer of suspension fibers (Figure 2C–E). Two cuticular apodemes, originating from the inner ridge of the pedicel, serve as anchoring point of the suspension fibers, therefore connecting the flagellum with the pedicel (Figure 2C). The Johnston’s organ (JO) of *P. spumarius* was composed of approximately 110 scolopidia arranged around the entire circumference of the pedicel lumen. Serial cross sections revealed that the scolopidia were organized in semicircular groups, symmetrically distributed along the inner wall of the pedicel (Figure 2E). All scolopidia were of the amphinematic type, i.e., with the sensory neurons that are directly attached to the cuticle. Each scolopidium consisted of two sensory neurons enveloped by three accessory cells: the scolopale cell (Sc), the attachment cell (Ac), and the glial cell (Gc) (Figure 3A–D). The sensory neurons were structurally similar, both presenting an axoneme with the typical 9 × 2 + 0 configuration of microtubules. Their dendrites projected distally into the scolopale space, where they were surrounded by the electron-dense extracellular scolopale tube secreted by the Sc (Figure 3A,B). The very distal part of each scolopidium was bent towards the central lumen, and was ultimately connected with the joint membrane, forming an aggregation of electron-dense material (Figure 2D–F). The ciliary region of the dendrites was supported by scolopale rods, composed of longitudinally oriented microtubules embedded in actin filaments (Figure 3C,D). Within this extracellular lumen, granular material surrounded the dendritic cilium (Figure 3A). At the distal level of the pedicel, the scolopidia established a firm anchoring with the apical region by means of the attachment cell. The Ac enclosed the distal part of the scolopale tube and extended towards the pedicel apex. The Ac terminated at the level of the suspension fibers, where the distal dendritic ends were anchored without penetrating the cuticle of the flagellum. In this way, the terminal portion of the scolopidia was embedded within the dense fibrous matrix, ensuring mechanical coupling between antennal movements and deformation of the dendritic cilia (Figure 2E,F).

The inner dendritic segments (Ids) of the two sensory neurons contained a double basal body. The proximal basal body was surrounded by ciliary rootlets that extended from the distal basal body and fused together near the sensory soma, forming a typical ciliary root (Figure 3F). The somata of the sensory neurons were located at the periphery of the antennal lumen, in close association with epidermal cells, and revealed large nuclei and associated glia cells (Figure 3E). The scolopale cell enveloped the sensory neurons from the level of the cell body up to the distal dendritic segments. Its proximal portion formed a complex labyrinth of cytoplasmic processes, while its distal portion secreted the scolopale rods. At the junction with the attachment cell, the Sc produced a mesaxon that enclosed the cilia within the scolopale lumen. The glial cell contributed to the general insulation and support of the scolopidium, occupying the periphery of the complex. The attachment cell, in addition to anchoring the scolopidium distally, also ensured continuity with the suspension fibers of the pedicel apex.

### 3.3. General Brain Anatomy

**Figure 4:**
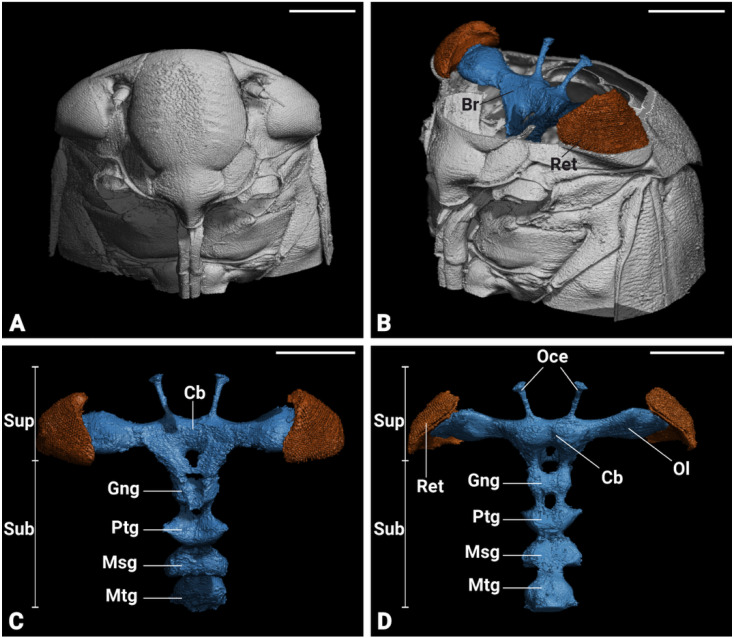
Micro-CT based 3D reconstruction of the head and brain in *P. spumarius*. (**A**) 3D Reconstruction of the head of *P. spumarius.* (**B**) Cross section of the head showing the brain (Br) within the internal lumen and retina (Ret) attached to the cuticle. (**C**,**D**) Ventral (**C**) and dorsal (**D**) view of *P. spumarius* brain showing the supraesophageal (Sup) and the subesophageal (Sub) ganglia. The supraesophageal ganglia revealed the central brain (Cb), retina (Ret), ocelli (Oce) and optic lobe (Ol). The subesophageal ganglia showed the gnathal ganglia (Gng) and three thoracic ganglia (prothoracic ganglion, Ptg; mesothoracic ganglion, Msg; metathoracic ganglion, Mtg). Scale bars: (**A**–**D**) = 500 μm.

**Figure 5:**
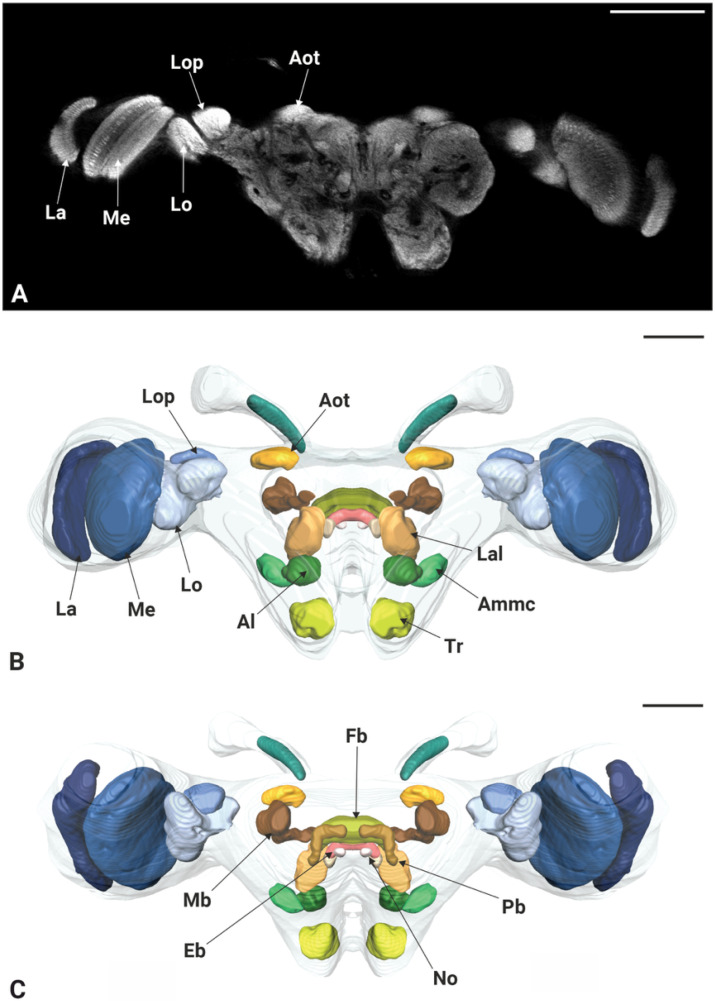
Immunocytochemistry-based 3D reconstruction of the brain of *P. spumarius.* (**A**) Single optical section through medial pat of the brain showing anti-synapsin staining. (**B**) Frontal view of *P. spumarius* brain with major neuropiles labeled. (**C**) Posterior view of the brain. Abbreviations: Al, antennal lobe; Ammc, antennal mechanosensory and motor center; Aot anterior optic tubercle; Eb, ellipsoid body; Fb, fan-shaped body; La, lamina; Lal, lateral accessory lobe; Lo, lobula; Lop, lobula plate; Mb, mushroom body; Me, medulla; No, Noduli, Pb, protocerebral bridge; Tr, tritocerebrum. Scale bars: (**A**) = 150 μm, (**B**,**C**) = 100 μm.

**Figure 6:**
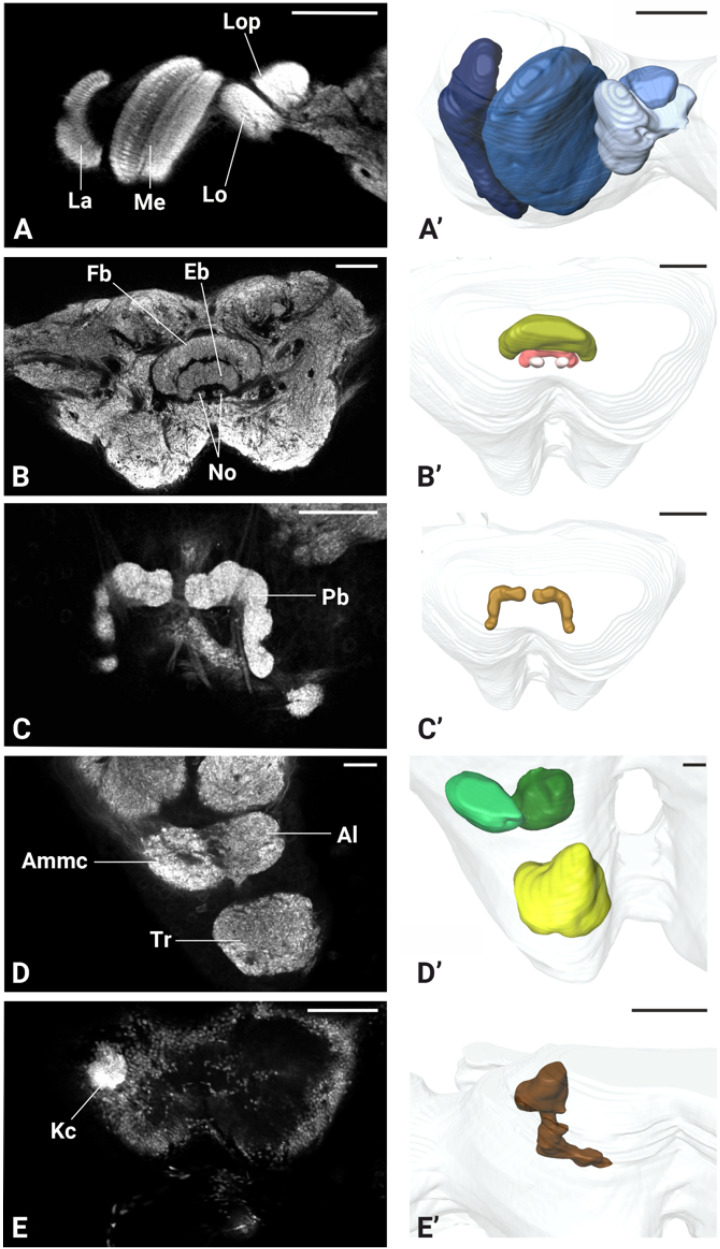
Optical sections and 3D renderings of neuropiles in the brain of *P. spumarius*. (**A**,**A’**) Optic lobe showing lamina (La), medula (Me), lobula (Lo) and lobula plate (Lop). (**B**,**B’**) Central brain and its 3D reconstruction, including the fan shaped body (Fb), ellipsoid body (Eb), and noduli (No). (**C**,**C’**) Protocerebral bridge (Pb) and its 3D reconstruction. (**D**,**D’**) Single section showing antennal lobe (Al), antennal mechanosensory and motor center (Ammc), and tritocerebrum (Tr) together with 3D reconstruction. (**E**,**E’**) Section showing dense nuclei of Kenyon cells (Kc) used as a landmark for the mushroom body. Scale bars: (**A**,**A’**) = 100 μm, (**B**,**B’**,**C**,**C’**) = 50 μm, (**D**,**D’**) = 20 μm, (**E**,**E’**) = 100 μm.

Micro-CT scans revealed the structural organization of *P. spumarius* brain, consisting of the central brain, sub-, and supraesophageal zones (Figure 4C,D). Brain regions appeared approximately symmetrical, with no visible distortion. The retina of the compound eye was firmly attached to the exoskeleton with no visible shrinkage (Figure 4B). The antennal nerve passed through the anterior tentorial pit and entered the brain dorso-laterally, below the optic lobes. Two ocelli were clearly distinguishable (Figure 4C,D). Although general brain staining with Lugol for Micro-CT was effective, it did not allow for the differentiation of brain regions. However, the immunohistological staining with synapsin revealed distinct neuropils (Figure 5A). We identified optic lobes (Ols), the anterior optic tubercle, central complex (Cx), lateral complex (Lx), antennal lobes (Als), antennal mechanosensory and motor center (Ammc), mushroom bodies (Mb), and tritocerebrum (Tb) (Figure 5 and Figure 6).

The protocerebrum of *P. spumarius* comprised the optic lobes and the central brain. The Ols subdivided into well-defined Lamina (La), Medulla (Me), and lobula complex (Lox) (Figure 5B,C and Figure 6A,A’,B,B’).

The La, the most distal optic neuropile, appeared as a narrow, elongated and curved structure, that extended over the Me. The Me was the largest optic neuropile, situated between the La and the Lox. The Lox lay medially to the Me and was further divided into the lobula (Lo) and lobula plate (Lop). The Lo was located anteriorly while the Lop was located posteriorly and appeared shallower (Figure 5B,C and Figure 6A,A’). The anterior optic tubercle (Aot) was intensively stained by anti-synapsin and was connected to Ols via the anterior optic tract (Figure 5B,C). Both sexes possessed a pair of ocelli, which were located antero-dorsally between the compound eyes. The Cx consisted of a group of neuropiles located at the center of the brain (Figure 5B,C). It included four substructures: central body (Cb), the protocerebral bridge (Pb), and the noduli (No) (Figure 6B,B’,C,C’). The central body was divided into upper central body, also known as fan-shaped body (Fb) in some insects, and lower central body or ellipsoid body (Eb). The Pb, the most posterior neuropile, consisted of two L bar-shaped hemispheres, which got closer to one another but never made direct contact (Figure 6C,C’). The Fb, the largest substructure of the Cx, was positioned dorsally and encapsulated the Eb. Below the Eb were the two No, the smallest structures within the Cx (Figure 6B,B’).

**Figure 7:**
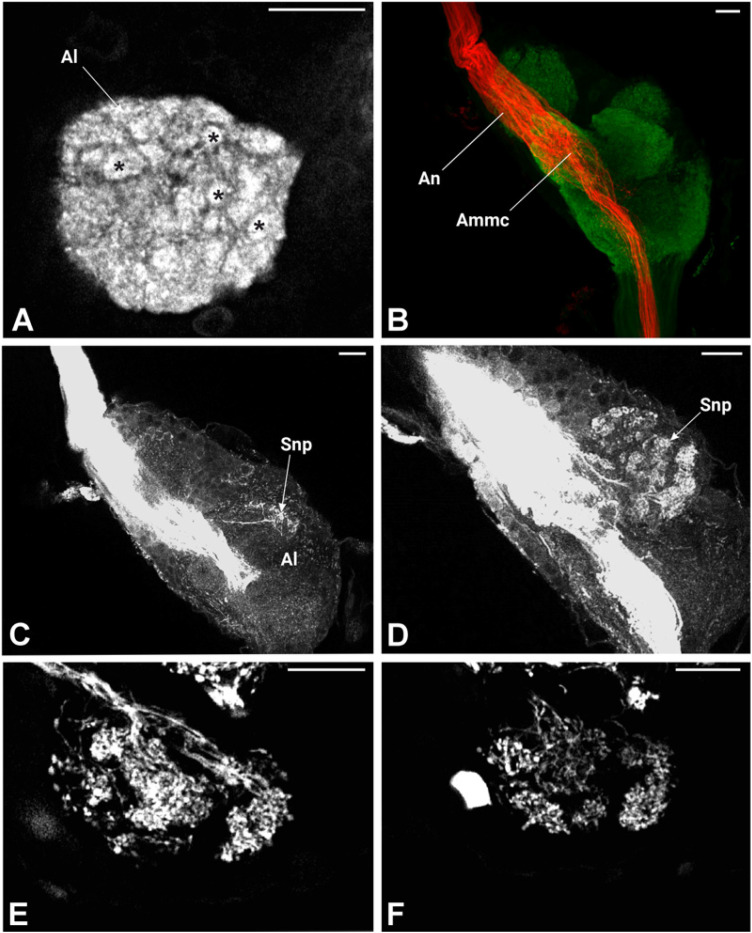
Antennal lobe and antennal nerve projection in the brain of *P. spumarius*. (**A**) Mid-level section of the antennal lobe (Al) showing numerous small, rounded glomeruli (*). (**B**) Antennal backfills staining revealing the antennal nerve (An) passing through the Ammc towards the tritocerebrum. (**C**,**D**) Sensory neuros projections (Snp) terminating in the antennal lobe (Al) shown via antennal nerve backfills. (**E**,**F**), Single sensillum backfills indicating sensory neurons arborization at different levels within the antennal lobe. Scale bars: (**A**–**F**) = 20 μm.

The lateral accessory lobe (Lal) was located laterally to the Eb and dorsally relative to the antennal lobe (Al). Mushroom body was less prominent neuropile. The calyx was identified by the intensive nuclei staining of the Kenyon cell (Kc) bodies, which appeared smaller in size when compared with nuclei of other parts of the brain (Figure 6E,E’). Pedunculus of the Mb was identified following synapsin-free regions of the brain connected to the calyx. The deutocerebrum of *P. spumarius* consisted of the antennal lobes (Als) and the antennal mechanosensory and motor center (Ammc) (Figure 6D,D’). The Al, the most anterior neuropile, was located medially next to the esophagus and appeared as hemispherical protrusions on the ventral brain surface. Al occupied 0.4% of the total brain volume and appeared to be made of numerous glomeruli (Figure 7A). However, their delimitation was not sufficient to enable us to carry out a 3D reconstruction of the Al. Adjacent to the Al lay the Ammc, which was positioned laterally and dorso-posteriorly to the Al.

**Figure 8:**
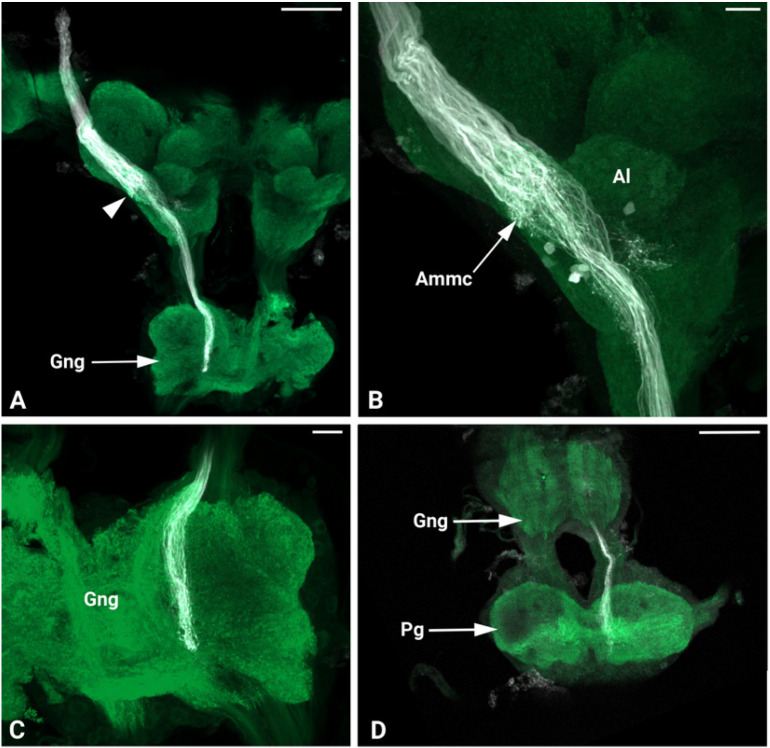
Maximum intensity projections of the confocal images of *P. spumarius* brain. (**A**) Overview of antennal nerve projections through the brain. A massive nerve staining is visible at the apical part of the brain, which becomes reduced when arborized at the Ammc level (arrow). At this level, the nerve projects to the gnathal ganglion (Gng). (**B**) Johnston’s organ afferents terminating at the Ammc, before continuing downward. (**C**) Johnston’s organ afferents innervating the gnathal ganglion (Gng) level. (**D**) Johnston’s organ nerve reaching and ending at the prothoracic ganglia (Ptg). Scale bars: (**A**) = 100 μm, (**B**,**C**) = 20 μm, (**D**) = 100 μm.

In *P. spumarius*, sensory neurons from the antenna projected into the brain via a prominent antennal nerve. The afferent projections were identified following the anterograde tracings applied at the distal part of the pedicel. The antennal nerve entered the brain near the lateral complex (Lx), with the majority of axons terminating in the Ammc (Figure 7B,C). From the Ammc, a subset of neurons projected into the Al (Figure 7D). Single sensillum backfills revealed individual neurons entering the Al and extensively arborizing throughout it, covering the majority of its surface (Figure 7E,F). No evidence of functional grouping was observed within the Al. Another subset of neurons bypassed the Ammc, projecting through the tritocerebrum to the gnathal ganglia (Gng) (Figure 8A,C,D). At the Gng, the primary outputs of the sensory neurons terminated. A small number of these neurons projected further, extending to and terminating in the prothoracic ganglia (Figure 8D).

## 4. Discussion

The present study revealed the ultrastructural organization of the Johnston’s organ in *P. spumarius* and provided an overview of its general brain organization. In many insects, intra- and interspecific communication signals are generated as mechanical stimuli resulting from abdomen trembling, wing spreading, stridulation, or drumming [13]. These signals are detected by chordotonal organs (such as tympanal organs and the Johnston’s organ) located in key positions, including the wing base, femur, ventral abdomen, larval abdominal wall, or antennae. The auditory organ consists of a series of scolopidia grouped into a larger structural unit—the Johnston’s organ [13,46,47].

The number of scolopidia that make up the Johnston’s organ varies across taxa. The most advanced Johnston’s organ, found in mosquitoes, consists of over 7000 scolopidia [48,49]. In contrast, *Periplaneta americana* has a Johnston’s organ with approximately 150 scolopidia, while in *P. spumarius*, we recorded 110 scolopidia. This number is comparable to those observed in related groups, such as Heteroptera and Sternorrhyncha (Aphididae), where the number of scolopidia typically does not exceed 50 [50,51]. However, among other closely related species, scolopidia counts vary, ranging from 25 in *Scaphoideus titanus* Ball, to 66 in *Hyalesthes obsoletus* Signoret, and 72 in *Metcalfa pruinosa* Say [52].

The complexity of the Johnston’s organ reflects the selective pressure for intra- and interspecific communication. In honeybees, the Johnston’s organ detects the vibrations generated during the waggle dance [3], while in *Drosophila melanogaster*, it responds to courtship signals produced by female wing vibrations. In planthoppers, the Johnston’s organ is involved in vibrational communication via substrate-borne signals.

In *P. spumarius*, we identified two types of scolopidia, distinguished by the number of sensory neurons they contain. This variation may suggest a potential multifunctional role for each type. The most common type contained two sensory neurons, while the scolopidia with one sensory neuron was less prevalent. Scolopidia with two sensory neurons are commonly observed in other insects, including aphids, *Periplaneta americana*, *Drosophila*, and mosquitoes [48,49,53,54]. In *Aedes aegypti*, this type of scolopidium is the most abundant and plays a key role in sound perception, accounting for 97% of the total scolopidia [48,49]. Recent physiological studies in *D. melanogaster* have shown that within these two-neuron scolopidia, one sensory cell is sensitive to vibrations, while the other responds to static deflections [55]. These findings support the hypothesis that one sensory neuron in the Johnston’s organ is specialized for hearing, while the other is involved in detecting gravity and wind. Unlike what has been described in other cicadellids, no central organ was detected in the antenna of *P. spumarius* [52].

In addition to the similarities in the ultrastructural organization of scolopidia, we aim to draw a parallel between the antennal organization and signal transmission mechanisms in *D. melanogaster* and *P. spumarius*. The antennae of *D. melanogaster* consist of three segments, with a feather-like arista located on the final segment, the funiculus [56]. In *Drosophila*, the arista and funiculus are the primary mechanotransducers, closely linked to one another. When sound particles or wind cause the displacement of the arista, it induces the rotation of the funiculus. This rotation generates a force on the scolopidia at the attachment point, stretching them and triggering mechanosensory cilia in the nerve cells. This process opens stretch-gated mechanotransduction channels, resulting in action potentials being transmitted along the antennal nerve [57,58,59].

A similar signal reception pathway likely occurs in *P. spumarius*, although with a key difference in antennal structure. In *Drosophila*, the arista is feather-like, while in *P. spumarius*, it is replaced by a long, thread-like filament. In *P. spumarius*, displacement of the flagellum by sound waves may transmit mechanical forces to the scolopidia of the Johnston’s organ, which are attached at the base of the flagellum, within the pedicel.

Apart the well-developed Johnston’s organ, a single sensillum campaniform and a single nerve at the base of the thread flagellum were observed. Taking into consideration the campaniform sensillum position (at the cuticular base of the flagellum), this sensillum could easily perceive slight flagellar movement, which could induce pressure on the sensillum. The direct pressure on the tip of the sensilla would result in its depolarization or, in contrast, hyperpolarization, similar to the response of scolopidia in *Drosophila* [60]. In addition, the presence of the sensory neuron at the flagellum base indicates a potential proprioceptive role of this structure.

The brain of *P. spumarius* followed the general organization found in the clade. However, when compared with the brain of true bugs (Hemiptera), it had less prominent Mb and low delimitation of the Al glomeruli [30]. Moreover, differences were found at the ganglia level, which appeared to be highly fused in aphids, whereas in *P*. *spumarius* they were separated by the short connectives [33].

Previous studies on the antennal lobe organization in Homoptera and Auchenorrhyncha have reported the presence of small, aglomerular or glomerular Als [33,34,36]. Our observations are consistent with these findings, as the Al in *P. spumarius* occupies approximately 0.4% of the total brain volume. A similar size has been reported for *Hyalesthes obsoletus*, where the Al constitutes about 0.6% of the brain [36]. This relatively small size stands in sharp contrast to the well-developed Als of commonly studied model organisms. For instance, in *Nasonia vitripennis* (Walker) (Pteromalidae), the Al accounts for 12% of the total brain volume [27]; in *Apis mellifera*, it represents approximately 5.7% [37]; in *Bombus terrestris* L., 5.2% [61]; and in *D. melanogaster*, around 5% [62,63]. Moreover, in *Apolygus lucorum* Meyer-Dür (Miridae), the Al occupies nearly 15% of the brain volume, making it the species with the most prominent Al reported to date [30].

The relatively small volume of the Al in the brain of *P. spumarius* likely reflects its sensory ecology. Indeed, the antennae of *P. spumarius* exhibit a significantly reduced sensory system, consisting of only 15 sensilla, of which just three are olfactory [39]. This low number of olfactory sensilla likely contributes to the small size of the glomeruli and the Al overall. A similarly reduced number of OSNs has been reported in other species, such as the psyllids and larvae of *D. melanogaster* [64,65].

No sexual dimorphism in Al size was observed. This is not surprising, as both sexes share a similar sensory system organization and ecological function. Differences in Al size between sexes have been reported in species where males and females exhibit distinct ecological behaviors, such as in the ant *Camponotus japonicus*. In this species, significant differences in Al size were found between males and unmated queens; however, no difference was observed between queens and workers [66].

Our staining of synapsin-rich neuropiles was largely successful. However, the glomeruli within the Als appeared poorly delimited, which prevented us from performing a 3D reconstruction. This low glomerular resolution may be associated with the presence of a thin glial sheath, which plays a crucial role in glomerular organization, as previously reported in Diptera [67].

Antennal backfill analysis revealed a prominent antennal nerve projecting into both the Ammc and the Al. Most of the fibers were associated with the Ammc, while only a few entered the Al. To further characterize the spatial organization of the Al, we performed single sensillum backfills. However, in addition to labelling sensory neurons from the sensilla basiconica, we also stained the nerve from the Johnston’s organ. To better visualize the sensilla and isolate individual sensilla, we extended the antennae up to the antennal ledge. This manipulation, however, led to the rupture of Johnston’s organ nerves. As a result, when neurobiotin was applied, it labelled both the targeted sensillum neurons and residual nerves from the Johnston’s organ. Despite this overlap, the staining clearly showed that the fibers entering the Al originated from the sensilla basiconica, and not from other sensillum types.

Our single sensillum backfills performed on sensilla basiconica revealed sensory neuron terminals arborizing across a large portion of the Al, without a clear functional subdivision in their arborization pattern. This diffuse localization may reflect the number of OSNs housed within each sensillum basiconicum and the total number of glomeruli. A previous study showed that each sensillum basiconicum contains 21 OSNs [39]. It is therefore conceivable that multiple OSNs from a single sensillum converge onto the same glomerulus. Similar findings have been reported in other species. In the mite *Phytoseiulus persimilis*, the peripheral olfactory system comprises five putative olfactory sensilla, with each glomerulus receiving input from a single sensory neuron [68]. A similar arrangement was also observed in the larvae of *D. melanogaster*, where 21 OSNs project to a single glomerulus [65]. However, in other insects, sensory neurons can be functionally arborized within the Al, receiving input from particular types of sensory neurons, such as olfactory or thermo-/hygroreceptive neurons [69,70,71,72] or receive inputs from multiple OSN [73].

The overall neural pattern of the Johnston’s organ afferents in *P. spumarius* is comparable with other species. In *Drosophila*, ants, and *Mythimna separata*, a major part of the Johnston’s organ projects to the Ammc [74,75,76]. In the earlier study on *Drosophila*, the Ammc was divided into five zones, depending on the spatial organization of the Johnston’s organ afferents [53,76,77,78,79]. Furthermore, each zone was associated with the specific function. Zones A and B receive inputs from the nerve cells responsible for the detection of near-sound and high-frequency vibrations while zones C and E are associated with the gravitational forces and wind-induced deflections. Contrary to *Drosophila* and *P. spumarius*, in honeybees, the afferents from Johnston’s organ are slightly different diverged in the brain [80]. Most of the afferents project to the posterior protocerebrum and subesophageal ganglion, while just a small number of afferents terminate in the Ammc.

Apart from the Ammc, the afferent neurons often project to other neuropiles. In *Drosophila* they are gnathal ganglia, wedge, thoracic-abdominal ganglia, anterior ventrolateral protocerebrum, saddle, and ventrolateral protocerebrum [81]. The former neuropile (ventrolateral protocerebrum) receives intensive inputs from the visual interneurons, thus is also known as a visual center in the central brain. Moreover, it receives inputs from the lateral horn. Therefore, the extension of the Johnston’s organ afferent to the ventrolateral protocerebrum may allow integration of both visual and mechanosensory signals, possibly improving continuous coordination during the flight by detecting wind currents [53,80].

Although the subesophageal ganglion is the primary center of gustatory neurons of the mouth parts, in *Drosophila* brain, the gustatory and Johnston’s organ neurons do not intersect. Thus, it is unlikely that these neurons have direct contact. Moreover, as the subesophageal ganglion houses terminals of the neurons derived from the thoracic and abdominal ganglia, it was proposed that this neuropile acts as an integration center for mechanosensory inputs from the Johnston’s organ and other parts of the brain [53].

In *P. spumarius* brain, we recorded an additional output center of the Johnston’s organ center, the prothoracic ganglia. This neuronal cell could simply be involved in transmitting information of different mechanosensory modalities to the prothoracic ganglia [78].

## 5. Conclusions

This study provides an integrated characterization of the Johnston’s organ and brain organization of *P. spumarius*. It reveals a complex mechanosensory system with approximately 110 scolopidia and a reduced olfactory system, reflected by the small and weakly glomerular Al. Neural tracing showed that most sensory neurons terminated in the Ammc, whereas only a few projected to the Al without forming clear functional arborizations. Additionally, several sensory neurons projected to both the sub- and supraesophageal zones. This study offers new insights into the functional adaptations that enable *P. spumarius* to detect environmental as well as inter- and intraspecific signals, thereby supporting the development of improved control strategies for this harmful pest.

## Data Availability

The original contributions presented in this study are included in the article. Further inquiries can be directed to the corresponding author.

## References

[1] Cocroft R.B., Rodríguez R.L. (2005). The Behavioral Ecology of Insect Vibrational Communication. BioScience.

[2] Goubaulr M., Batchelor T.P., Romani R., Linforth R.S.T., Fritzsche M., Francke W., Hardy I.C.W. (2008). Volatile Chemical Release by Bethylid Wasps: Identity, Phylogeny, Anatomy and Behaviour. Biol. J. Linn. Soc..

[3] Ai H., Kai K., Kumaraswamy A., Ikeno H., Wachtler T. (2017). Interneurons in the Honeybee Primary Auditory Center Responding to Waggle Dance-like Vibration Pulses. J. Neurosci..

[4] Saitta V., Rebora M., Piersanti S., Salerno G. (2024). Visual and Chemical Cues in the Host Plant Selection of the Melon Ladybird *Chnootriba elaterii* (Coleoptera: Coccinellidae). Arthropod-Plant Interact..

[5] Zacharuk R.Y., Kerkut G.A., Gilbert L.I. (1985). Antennae and Sensilla. Comprehensive Insect Physiology, Chemistry and Pharmacology.

[6] Keil T.A., Hansson B.S. (1999). Morphology and Development of the Peripheral Olfactory Organs. Insect Olfaction.

[7] Romani R., Isidoro N., Bin F. (2010). Antennal Structures Used in Communication by Egg Parasitoids. Egg Parasitoids in Agroecosystems with Emphasis on Trichogramma.

[8] Dippel S., Kollmann M., Oberhofer G., Montino A., Knoll C., Krala M., Rexer K.-H., Frank S., Kumpf R., Schachtner J. (2016). Morphological and Transcriptomic Analysis of a Beetle Chemosensory System Reveals a Gnathal Olfactory Center. BMC Biol..

[9] Galizia C.G., Rössler W. (2010). Parallel Olfactory Systems in Insects: Anatomy and Function. Annu. Rev. Entomol..

[10] Masse N.Y., Turner G.C., Jefferis G.S.X.E. (2009). Olfactory Information Processing in *Drosophila*. Curr. Biol..

[11] Vosshall L.B., Stocker R.F. (2007). Molecular Architecture of Smell and Taste in *Drosophila*. Annu. Rev. Neurosci..

[12] Yack J.E. (2004). The Structure and Function of Auditory Chordotonal Organs in Insects. Microsc. Res. Tech..

[13] Field L.H., Matheson T. (1998). Chordotonal Organs of Insects. Adv. Insect Phys..

[14] Kavlie R.G., Albert J.T. (2013). Chordotonal Organs. Curr. Biol..

[15] Göpfert M.C., Robert D. (2000). Nanometre-Range Acoustic Sensitivity in Male and Female Mosquitoes. Proc. R. Soc. B Biol. Sci..

[16] Krishnan A., Sane S.P. (2015). Antennal Mechanosensors and Their Evolutionary Antecedents. Advances in Insect Physiology.

[17] Matsuo E., Kamikouchi A. (2013). Neuronal Encoding of Sound, Gravity, and Wind in the Fruit Fly. J. Comp. Physiol. A Neuroethol. Sens. Neural Behav. Physiol..

[18] Sun Y., Liu L., Ben-Shahar Y., Jacobs J.S., Eberl D.F., Welsh M.J. (2009). TRPA Channels Distinguish Gravity Sensing from Hearing in Johnston’s Organ. Proc. Natl. Acad. Sci. USA.

[19] Anton S., Homberg U. (1999). Antennal Lobe Structure. Insect Olfaction.

[20] Grabe V., Baschwitz A., Dweck H.K.M., Lavista-Llanos S., Hansson B.S., Sachse S. (2016). Elucidating the Neuronal Architecture of Olfactory Glomeruli in the *Drosophila* Antennal Lobe. Cell Rep..

[21] Strausfeld N.J. (2012). Arthropod Brains: Evolution, Functional Elegance, and Historical Significance.

[22] Dekker T., Ibba I., Siju K.P., Stensmyr M.C., Hansson B.S. (2006). Olfactory Shifts Parallel Superspecialism for Toxic Fruit in *Drosophila melanogaster* Sibling, *D. sechellia*. Curr. Biol..

[23] Ibba I., Angioy A.M., Hansson B.S., Dekker T. (2010). Macroglomeruli for Fruit Odors Change Blend Preference in *Drosophila*. Naturwissenschaften.

[24] Bressel O.C., Khan M., Mombaerts P. (2016). Linear Correlation between the Number of Olfactory Sensory Neurons Expressing a given Mouse Odorant Receptor Gene and the Total Volume of the Corresponding Glomeruli in the Olfactory Bulb. J. Comp. Neurol..

[25] Acebes A., Ferrús A. (2001). Increasing the Number of Synapses Modifies Olfactory Perception in *Drosophila*. J. Neurosci..

[26] Galizia C.G., McIlwrath S.L., Menzel R. (1999). A Digital Three-Dimensional Atlas of the Honeybee Antennal Lobe Based on Optical Sections Acquired by Confocal Microscopy. Cell Tissue Res..

[27] Groothuis J., Pfeiffer K., el Jundi B., Smid H.M. (2019). The Jewel Wasp Standard Brain: Average Shape Atlas and Morphology of the Female *Nasonia vitripennis* Brain. Arthropod Struct. Dev..

[28] Laissue P.P., Reiter C., Hiesinger P.R., Halter S., Fischbach K.F., Stocker R.F. (1999). Three-Dimensional Reconstruction of the Antennal Lobe in *Drosophila melanogaster*. J. Comp. Neurol..

[29] Solari P., Corda V., Sollai G., Kreissl S., Galizia C.G., Crnjar R. (2016). Morphological Characterization of the Antennal Lobes in the Mediterranean Fruit Fly *Ceratitis capitata*. J. Comp. Physiol. A Neuroethol. Sens. Neural Behav. Physiol..

[30] Xie G.Y., Ma B.W., Liu X.L., Chang Y.J., Chen W.B., Li G.P., Feng H.Q., Zhang Y.J., Berg B.G., Zhao X.C. (2019). Brain Organization of *Apolygus lucorum*: A Hemipteran Species with Prominent Antennal Lobes. Front. Neuroanat..

[31] Ignell R., Anton S., Hansson B.S. (2001). The Antennal Lobe of Orthoptera—Anatomy and Evolution. Brain Behav. Evol..

[32] Schachtner J., Schmidt M., Homberg U. (2005). Organization and Evolutionary Trends of Primary Olfactory Brain Centers in Tetraconata (Crustacea+Hexapoda). Arthropod Struct. Dev..

[33] Kollmann M., Minoli S., Bonhomme J., Homberg U., Schachtner J., Tagu D., Anton S. (2011). Revisiting the Anatomy of the Central Nervous System of a Hemimetabolous Model Insect Species: The Pea Aphid *Acyrthosiphon pisum*. Cell Tissue Res..

[34] Kristoffersen L., Hansson B.S., Anderbrant O., Larsson M.C. (2008). Aglomerular Hemipteran Antennal Lobes—Basic Neuroanatomy of a Small Nose. Chem. Senses.

[35] Rebora M., Piersanti S., Salerno G., Gorb S. (2015). The Antenna of a Burrowing Dragonfly Larva, *Onychogomphus forcipatus* (Anisoptera, Gomphidae). Arthropod Struct. Dev..

[36] Rossi Stacconi M.V., Hansson B.S., Rybak J., Romani R. (2014). Comparative Neuroanatomy of the Antennal Lobes of 2 Homopteran Species. Chem. Senses.

[37] Brandt R., Rohlfing T., Rybak J., Krofczik S., Maye A., Westerhoff M., Hege H.C., Menzel R. (2005). Three-Dimensional Average-Shape Atlas of the Honeybee Brain and Its Applications. J. Comp. Neurol..

[38] Saponari M., Boscia D., Nigro F., Martelli G.P. (2013). Identification of DNA Sequences Related to *Xylella fastidiosa* in Oleander, Almond and Olive Trees Exhibiting Leaf Scorch Symptoms in Apulia (Southern Italy). J. Plant Pathol..

[39] Ranieri E., Ruschioni S., Riolo P., Isidoro N., Romani R. (2016). Fine Structure of Antennal Sensilla of the Spittlebug *Philaenus spumarius* L. (Insecta: Hemiptera: Aphrophoridae). I. Chemoreceptors and Thermo-/Hygroreceptors. Arthropod Struct. Dev..

[40] Anastasaki E., Psoma A., Partsinevelos G., Papachristos D., Milonas P. (2021). Electrophysiological Responses of *Philaenus spumarius* and *Neophilaenus campestris* Females to Plant Volatiles. Phytochemistry.

[41] Cascone P., Quarto R., Iodice L., Cencetti G., Formisano G., Spiezia G., Giorgini M., Michelozzi M., Guerrieri E. (2022). Behavioural Response of the Main Vector of *Xylella fastidiosa* towards Olive VOCs. Entomol. Gen..

[42] Ganassi S., Cascone P., Di Domenico C., Pistillo M., Formisano G., Giorgini M., Grazioso P., Germinara G.S., De Cristofaro A., Guerrieri E. (2020). Electrophysiological and Behavioural Response of *Philaenus spumarius* to Essential Oils and Aromatic Plants. Sci. Rep..

[43] Sevarika M., Rondoni G., Ganassi S., Pistillo O.M., Germinara G.S., De Cristofaro A., Romani R., Conti E. (2022). Behavioural and Electrophysiological Responses of *Philaenus spumarius* to Odours from Conspecifics. Sci. Rep..

[44] Sevarika M., Di Giulio A., Rondoni G., Conti E., Romani R. (2022). Morpho-Functional Analysis of the Head Glands in Three *Auchenorrhyncha* Species and Their Possible Biological Significance. Microsc. Microanal..

[45] Avosani S., Daher E., Franceschi P., Ciolli M., Verrastro V., Mazzoni V. (2020). Vibrational Communication and Mating Behavior of the Meadow Spittlebug *Philaenus spumarius*. Entomol. Gen..

[46] Keil T.A. (1997). Functional Morphology of Insect Mechanoreceptors. Microsc. Res. Tech..

[47] McIver S.B. (1985). Mechanoreception. Comprehensive Insect Physiology, Biochemistry and Phramacology.

[48] Boo K.S., Richards A.G. (1975). Fine Structure of the Scolopidia in the Johnston’s Organ of Male *Aedes aegypti* (L.) (Diptera: Culicidae). Int. J. Insect Morphol. Embryol..

[49] Boo K.S., Richards A.G. (1975). Fine Structure of Scolopidia in Johnston’s Organ of Female *Aedes aegypti* Compared with That of the Male. J. Insect Physiol..

[50] Bromley A.K., Dunn J.A., Anderson M. (1980). Ultrastructure of the Antennal Sensilla of Aphids. Cell Tissue Res..

[51] Jeram S., Pabst M.A. (1996). Johnston’s Organ and Central Organ in *Nezara viridula* (L.) (Heteroptera, Pentatomidae). Tissue Cell.

[52] Rossi Stacconi M.V., Romani R. (2013). The Johnston’s Organ of Three Homopteran Species: A Comparative Ultrastructural Study. Arthropod Struct. Dev..

[53] Kamikouchi A., Shimada T., Ito K. (2006). Comprehensive Classification of the Auditory Sensory Projections in the Brain of the Fruit Fly *Drosophila melanogaster*. J. Comp. Neurol..

[54] Toh Y. (1981). Fine Structure of Sense Organs on the Antennal Pedicel and Scape of the Male Cockroach, *Periplaneta americana*. J. Ultrasruct. Res..

[55] Ishikawa Y., Fujiwara M., Wong J., Ura A., Kamikouchi A. (2020). Stereotyped Combination of Hearing and Wind/Gravity-Sensing Neurons in the Johnston’s Organ of Drosophila. Front. Physiol..

[56] Foelix R.F., Stocker R.F., Steinbrecht R.A. (1989). Fine Structure of a Sensory Organ in the Arista of *Drosophila melanogaster* and Some Other Dipterans. Cell Tissue Res..

[57] Eberl D.F. (1999). Feeling the Vibes: Chordotonal Mechanisms in Insect Hearing. Curr. Opin. Neurobiol..

[58] Göpfert M.C., Robert D. (2002). The Mechanical Basis of *Drosophila* Audition. J. Exp. Biol..

[59] Todi S.V., Sharma Y., Eberl D.F. (2004). Anatomical and Molecular Design of the *Drosophila* Antenna as a Flagellar Auditory Organ. Microsc. Res. Tech..

[60] Pézier A., Blagburn J.M. (2013). Auditory Responses of Engrailed and Invected-Expressing Johnston’s Organ Neurons in *Drosophila Melanogaster*. PLoS ONE.

[61] Rother L., Kraft N., Smith D.B., el Jundi B., Gill R.J., Pfeiffer K. (2021). A Micro-CT-Based Standard Brain Atlas of the Bumblebee. Cell Tissue Res..

[62] Rein K., Zöckler M., Mader M.T., Grübel C., Heisenberg M. (2002). The *Drosophila* Standard Brain. Curr. Biol..

[63] Shao H.C., Wu C.C., Chen G.Y., Chang H.M., Chiang A.S., Chen Y.C. (2014). Developing a Stereotypical *Drosophila* Brain Atlas. IEEE Trans. Biomed. Eng..

[64] Kristoffersen L., Hallberg E., Wallén R., Anderbrant O. (2006). Sparse Sensillar Array on *Trioza apicalis* (Homoptera, Triozidae) Antennae-an Adaptation to High Stimulus Levels?. Arthropod Struct. Dev..

[65] Ramaekers A., Magnenat E., Marin E.C., Gendre N., Jefferis G.S.X.E., Luo L., Stocker R.F. (2005). Glomerular Maps without Cellular Redundancy at Successive Levels of the *Drosophila* Larval Olfactory Circuit. Curr. Biol..

[66] Nishikawa M., Nishino H., Misaka Y., Kubota M., Tsuji E., Satoji Y., Ozaki M., Yokohari F. (2008). Sexual Dimorphism in the Antennal Lobe of the Ant *Camponotus japonicus*. Zool. Sci..

[67] Stocker R.F., Lienhard M.C., Borst A., Fischbach K.F. (1990). Neuronal Architecture of the Antennal Lobe in *Drosophila melanogaster*. Cell Tissue Res..

[68] Van Wijk M., Wadman W.J., Sabelis M.W. (2006). Morphology of the Olfactory System in the Predatory Mite *Phytoseiulus persimilis*. Exp. Appl. Acarol..

[69] Hansson B.S., Christensen T.A., Hildebrand J.G. (1991). Functionally Distinct Subdivisions of the Macroglomerular Complex in the Antennal Lobe of the Male Sphinx Moth *Manduca sexta*. J. Comp. Neurol..

[70] Kanzaki R., Soo K., Seki Y., Wada S. (2003). Projections to Higher Olfactory Centers from Subdivisions of the Antennal Lobe Macroglomerular Complex of the Male Silkmoth. Chem. Senses.

[71] Kleineidam C.J., Obermayer M., Halbich W., Rössler W. (2005). A Macroglomerulus in the Antennal Lobe of Leaf-Cutting Ant Workers and Its Possible Functional Significance. Chem. Senses.

[72] Ruchty M., Helmchen F., Wehner R., Kleineidam C.J. (2010). Representation of Thermal Information in the Antennal Lobe of Leaf-Cutting Ants. Front. Behav. Neurosci..

[73] Bicker G., Stern M. (2020). Structural and Functional Plasticity in the Regenerating Olfactory System of the Migratory Locust. Front. Physiol..

[74] Grob R., Tritscher C., Grübel K., Stigloher C., Groh C., Fleischmann P.N., Rössler W. (2021). Johnston’s Organ and Its Central Projections in *Cataglyphis* Desert Ants. J. Comp. Neurol..

[75] Ma B.W., Zhao X.C., Berg B.G., Xie G.Y., Tang Q.B., Wang G.R. (2017). Central Projections of Antennal and Labial Palp Sensory Neurons in the Migratory Armyworm *Mythimna Separata*. Front. Cell Neurosci..

[76] Patella P., Wilson R.I. (2018). Functional Maps of Mechanosensory Features in the *Drosophila* Brain. Curr. Biol..

[77] Kamikouchi A., Inagaki H.K., Effertz T., Hendrich O., Fiala A., Göpfert M.C., Ito K. (2009). The Neural Basis of *Drosophila* Gravity-Sensing and Hearing. Nature.

[78] Matsuo E., Yamada D., Ishikawa Y., Asai T., Ishimoto H., Kamikouchi A. (2014). Identification of Novel Vibration- and Deflection-Sensitive Neuronal Subgroups in Johnston’s Organ of the Fruit Fly. Front. Physiol..

[79] Yorozu S., Wong A., Fischer B.J., Dankert H., Kernan M.J., Kamikouchi A., Ito K., Anderson D.J. (2009). Distinct Sensory Representations of Wind and Near-Field Sound in the *Drosophila* Brain. Nature.

[80] Ai H., Nishino H., Itoh T. (2007). Topographic Organization of Sensory Afferents of Johnston’s Organ in the Honeybee Brain. J. Comp. Neurol..

[81] Matsuo E., Seki H., Asai T., Morimoto T., Miyakawa H., Ito K., Kamikouchi A. (2016). Organization of Projection Neurons and Local Neurons of the Primary Auditory Center in the Fruit Fly *Drosophila melanogaster*. J. Comp. Neurol..

